# The effect of a telephone follow-up call for older patients, discharged home from the emergency department on health-related outcomes: a systematic review of controlled studies

**DOI:** 10.1186/s12245-021-00336-x

**Published:** 2021-02-18

**Authors:** Merel van Loon-van Gaalen, Britt van Winsen, M. Christien van der Linden, Jacobijn Gussekloo, Roos C. van der Mast

**Affiliations:** 1 Emergency Department, Haaglanden Medical Center, P.O. Box 432, 2501 CK The Hague, The Netherlands; 2 Department of Internal Medicine, Section of Gerontology and Geriatrics, Leiden University Medical Center, Leiden, The Netherlands; 3 Department of Public Health and Primary Care, Leiden University Medical Center, Leiden, The Netherlands; 4 Department of Psychiatry, Leiden University Medical Center, Leiden, The Netherlands; 5 Department of Psychiatry, CAPRI-University, Antwerp, Belgium

**Keywords:** Older patients, Emergency department, Telephone, Postdischarge follow-up, Geriatric

## Abstract

**Background:**

Older patients discharged from the emergency department (ED) are at increased risk for adverse outcomes. Transitional care programs offer close surveillance after discharge, but are costly. Telephone follow-up (TFU) may be a low-cost and feasible alternative for transitional care programs, but its effects on health-related outcomes are not clear.

**Aim:**

We systematically reviewed the literature to evaluate the effects of TFU by health care professionals after ED discharge to an unassisted living environment on health-related outcomes in older patients compared to controls.

**Methods:**

We conducted a multiple electronic database search up until December 2019 for controlled studies examining the effects of TFU by health care professionals for patients aged ≥65 years, discharged to an unassisted living environment from a hospital ED. Two reviewers independently assessed eligibility and risk of bias.

**Results:**

Of the 748 citations, two randomized controlled trials (including a total of 2120 patients) met review selection criteria. In both studies, intervention group patients received a scripted telephone intervention from a trained nurse and control patients received a patient satisfaction survey telephone call or usual care. No demonstrable benefits of TFU were found on ED return visits, hospitalization, acquisition of prescribed medication, and compliance with follow-up appointments. However, many eligible patients were not included, because they were not reached or refused to participate.

**Conclusions:**

No benefits of a scripted TFU call from a nurse were found on health services utilization and discharge plan adherence by older patients after ED discharge. As the number of high-quality studies was limited, more research is needed to determine the effect and feasibility of TFU in different older populations.

PROSPERO registration number CRD42019141403.

**Supplementary Information:**

The online version contains supplementary material available at 10.1186/s12245-021-00336-x.

## Introduction

### Background

Older patients discharged from the emergency department (ED) are at increased risk of functional decline, ED return visits, hospitalization and death [1–5]. Risk factors associated with these outcomes are pre-existing functional and cognitive impairment, but also lack of social support, living alone, and feeling depressed [1, 6]. Therefore, older patients discharged home from the ED may need close medical surveillance and adequate care transition from the ED to home.

In the last decades, many transitional care programs were started with the aim of preventing and reducing problems after discharge from the ED and limiting ED return visits and hospitalization. Most transitional care programs focus on older high-risk patients, detected by geriatric assessment. These programs consist of discharge arrangements for community services and patient-education, which usually start during the patients’ ED stay and are continued afterwards, either by home visits, telephone calls, or both [1, 7, 8].

Several studies examining the effect of these transitional care programs found some positive effects, e.g., reduction in ED return visits [9], hospital admissions [10], and nursing home admissions [11]. However, many of these programs proved to be time-consuming and therefore involved deployment of additional staff, leading to considerable personnel costs [9, 12]. This may be beyond the ability of many EDs to implement.

As an alternative intervention, telephone follow-up (TFU) is described as an inexpensive and easy to organize method of post-discharge care in various medical populations and settings [13–16]. Feasibility has been demonstrated in multiple medical settings, including the ED. [17–19] However, previous systematic reviews examining the effect of TFU by hospital-based and primary care professionals after hospital admission in (adult) patients of all ages found inconclusive evidence about the effects of TFU. The authors of the reviews reported a large variety in study methods and outcome measures and low methodological quality of the included studies [13, 20, 21]. The effect of TFU in older patients discharged home from the ED has not yet been examined in a systematic review, apart from one “short-cut review,” solely focusing on compliance with follow-up visits and discharge instructions [22]. The effects of TFU in older adults, discharged from the ED, on other outcomes, like ED return visits and hospitalization, are still unknown.

### Aim

The aim of this systematic review of controlled studies was to determine the effects of a telephone follow-up (TFU) call from a health care professional for older patients after discharge from the ED to an unassisted living environment on health-related and patient-oriented outcomes, including ED return visits and hospitalization, but also compliance with discharge instructions, general functioning, patient satisfaction, and emotional wellbeing.

## Methods

This systematic review was done following the Preferred Reporting Items for Systematic Reviews and Meta-Analyses (PRISMA) guidelines [23, 24].

### Protocol and registration

A protocol describing the research question, search strategy, in- and exclusion criteria, and methods of the analysis was made in advance and registered in PROSPERO (registration number: CRD42019141403).

### Search strategy and selection criteria

We performed an electronic search of MEDLINE, Ovid EMBASE, and the Wiley Cochrane Library in the Cochrane Database of Systematic Reviews and Central Register of Controlled Trials from the beginning of indexing until December 1, 2019. Search terms used were a combination of Medical Subject Heading terms and relevant keywords; no restriction with respect to language was used. Full details of the search strategy are available in Additional file 1. Detailed selection criteria are described in Table 1.

**Table 1:** Selection criteria

Category	Inclusion criteria	Exclusion criteria
Population	Patients aged 65 years and older, discharged from the ED to an unassisted living environment.	Patients aged under 65 years; Patients discharged from the ED to an assisted living environment
Intervention	Telephone follow-up call by health care professional after ED discharge	Any other kind of transitional care; Telephone follow-up not conducted as independent intervention; Telephone follow-up calls by others than health care professionals.
Control condition	Usual care or patient satisfaction survey telephone call	
Outcome measures	Any health-related, patient-oriented outcome, including:	Outcomes not health-related or patient-oriented
	*Health services utilization*, including ED return visits, hospitalization, follow-up visits	
	*Physical health outcomes*, including level of activities of daily living, independence	
	*Psychosocial health outcomes*, including quality of life, mood, satisfaction	
	*Other patient-oriented outcomes*, including treatment adherence, knowledge of disease and symptom management	
Setting	Discharged from hospital-based ED	Discharged from hospital ward or primary care setting
Study type	Case-control or (randomized) controlled clinical trials	Uncontrolled studies

Besides searching in electronic databases, we hand-searched several clinical trial websites (presented in Additional file 2) to identify relevant unpublished and ongoing research and publications in journals that are not peer-reviewed. Reference lists of selected full-text articles were hand-searched for other potentially relevant articles. Original, full-text articles with case-control or (randomized) controlled clinical trial design were eligible for inclusion.

### Study selection

Two investigators (MvL and BvW) independently screened the electronic search results on title and abstract to identify potentially relevant articles, according to the predefined selection criteria (see Table 1). Disagreements concerning which citations were suitable for full-text review were resolved by discussion in the presence of a third author (MCvdL) until consensus was achieved. In case of disagreement, the full text of the article was retrieved and reviewed. Full-text articles of relevant citations were reviewed independently by two investigators (MvL and BvW). Agreement about which articles were suitable for inclusion was again achieved by discussion in the presence of the third author (MCvdL). Records were managed using ® 2020 Mendeley Ltd.

### Risk of bias assessment

Using the Cochrane risk of a bias tool, two reviewers (MvL and MCvdL) independently assessed the risk of bias for each individual study on seven domains (Additional file 3) [25].

### Data extraction and synthesis

We developed a data extraction sheet, based on the Cochrane Consumers and Communication Review Group’s data extraction template (see Additional file 3).

One reviewer (MvL) independently extracted data on patient and study characteristics and another reviewer (MCvdL) checked the extracted data on the sheets. Disagreements were resolved by discussion until consensus was reached. We contacted the author of the two included studies for further information concerning the methods, blinding of research staff and numerical outcome data. The author did not respond and hence the questions we had could not be clarified.

## Results

### Study selection

**Fig. 1:**
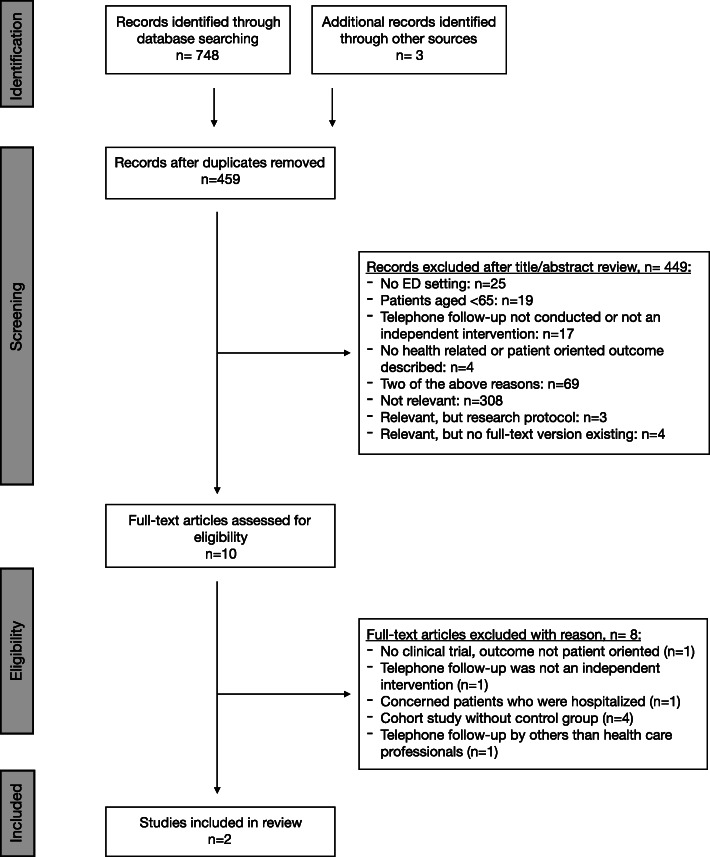
Flow diagram of study selection. n number, ED emergency department

Of the 748 citations until December 1, 2019, only two studies met the selection criteria for our systematic analysis (Fig. 1). Searching clinical trial websites did not yield any relevant ongoing unpublished research.

### Overview of included studies

Table 2 summarizes the study characteristics and outcome measures of the two included studies. Both studies were single-centred randomized controlled trials (RCTs) from the same author, performed in the same academic ED, but with a different study population in a different study period [26, 27].

**Table 2:** Characteristics, outcome measures, and feasibility of included studies

Characteristics	Biese et.al. 2014 [26]	Biese et.al. 2018 [27]
Setting, country	Academic center ED, USA	Academic center ED, USA
Study design	RCT	RCT
Aim(s)	To investigate whether an ED postdischarge telephone intervention by nurse improves discharge plan adherence.	To investigate whether an ED postdischarge telephone intervention by call-center nurse decreases ED return visit rates, hospitalization or death within 30 days after ED visit.
Study period	From September 5 until November 9, 2010	From August 2013 to March 2016
Study patients	Patients ≥65 years, discharged home from ED	Patients ≥65 years, discharged home from ED
Recruitment of study patients	Randomization before first call Recruitment by telephone after mental cognition screening examination was passed and informed consent was obtained.	Recruitment by telephone after mental cognition screening examination was passed and informed consent was obtained. Subsequent randomization.
Description of intervention: intervention group	Telephone call following pre-written script from trained study nurse within 1–3 days after ED discharge to review discharge instructions and offer assistance with discharge plan compliance.	Telephone call following pre-written script from call-center nurse within 1–3 days after ED discharge to identify problems, review discharge instructions and offer assistance with discharge plan compliance, advice if not feeling well.
Description of intervention: control group(s)	*Placebo group:* scripted patient satisfaction survey telephone call from research assistant 1–3 days after ED discharge. *Control group:* no telephone intervention	Scripted patient satisfaction survey telephone call from call-center nurse 1–3 days after ED discharge.
Sample size	Intervention group: *n*=39; placebo group: *n*=35; control group: *n*=46	Intervention group: *n*=999; control group: *n*=1001
Outcome measures	Primary outcome measures: Scheduled physician appointment within 5 days. Filled medication prescription. Knowledge of name, dosage, indication of prescribed medication.	Primary outcome measures: Days from ED discharge to ED return visit, 30-day hospitalization or death.
	Secondary outcome measures: 35-day hospitalization 35-day ED return visits	Secondary outcome measures: Scheduled physician appointment within 30 days. Difficulty acquiring prescribed medication.
Results of outcome measures	Primary outcome measures: Physician appointment ≤ 5 days: 54% (I), 20% (P), 37%(C); *p*=0.04 Filled prescription: 96% (I), 94%(P), 94% (C); NS. Knowledge name/dosage of medication: 92%(I), 94%(P), 89%(C); NS. Knowledge of reason for medication: 96%(I), 100% (P), 100%(C); NS.	Primary outcome measures: ED return visits ≤30 days: 12.2% (I) vs. 12.5% (C); NS. Hospitalization ≤30 days: 9.0% (I) vs. 7.4%(C); NS. Death ≤30 days: 0%(I) vs. 0.51% (C); NS.
	Secondary outcome measures: ED return visits/hospitalization ≤35 days: 22%(I), 33%(P), 27%(C); NS.	Secondary outcome measures: Physician appointment ≤ 30 days: 80.8% (I) vs. 80.8%(C); NS. Difficulty acquiring medication: 15.5% (I) vs. 15.6%(C); NS.
Feasibility	178 eligible patients: 120 (67%) included, 18 (10%) declined and 19 (11%) not reached during follow-up. 12 (7%) Were disqualified from primary outcome analysis, because of return to ED or other hospital setting within 5 days. Three were excluded for other reasons. Six had incomplete surveys. No patients failed mental screening examination. Inclusions only on Sunday, Monday, Tuesday, and not more than 9 inclusions per day.	Of the 6463 eligible patients, 2000 (31%) consented to participate. 2712 (42%) Patients were not reached, 1683 (45%) patients who were reached declined participation, 37 were lost on call back and 31 failed mental screening examination. Inclusions 24/7.

*C* control group, *ED* emergency department, *I* intervention group, *NS* not significant (*p*>0.05), *P* placebo group, *RCT* randomized controlled trial, *USA* United States of America

The studies involved a total of 2120 patients aged ≥ 65 years who were discharged home from the ED. Study sample sizes were 120 and 2000 patients, respectively. The duration of follow-up ranged from 30 to 35 days. In both studies, trained nurses recruited patients by telephone. Older patients or, if they were not available, their caregivers or spouses, had to pass a mental cognition screening examination before participation. Patients in the intervention group received a post-discharge telephone intervention in which they were surveyed about their wellbeing, understanding of their ED diagnoses, discharge instructions, follow-up appointments, and management of medications. The nurse provided review and re-emphasis of discharge instructions, reinforcement of follow-up appointments, assistance in making appointments, and advice if not feeling well. Control group patients received either a telephone call during which satisfaction with their care during the ED visit was assessed, or no telephone call after discharge. One study (Biese et al. 2014) compared the outcomes of three patient groups: an intervention group, a placebo group in which patients received a patient satisfaction survey telephone call, and a control group in which patients received no telephone call after discharge. The primary objective of this study was to investigate whether TFU improved discharge plan adherence [26]. The second study (Biese et al. 2018) consisted of two patients groups: an intervention group in which patients received an intervention telephone call and a control group in which patients received a patient satisfaction survey telephone call. The primary outcome measures of the study were the rates of ED return visits, hospital admissions, or death within 30 days after ED discharge. Only this study was of sufficient sample size to detect a significant difference on these outcome measures between the study groups [27].

### Risk of bias assessment of included studies

In both studies, the randomization process was well performed and described, ensuring a low risk of selection bias. Patients were not aware of the interventions, but blinding of personnel was not possible. However, the telephone calls were scripted in order to prevent performance bias. It was unclear whether the nurses who performed the data collection calls were (completely) blinded, but these telephone calls were also scripted to prevent detection bias. Loss to follow-up and incomplete data of included patients was limited. Methods were followed and expected outcomes were reported as planned in previously published study protocols. In the first study of Biese et al., it was not clear whether patients were analyzed according to intention to treat [26]. In both studies, patients who did not pass the mental cognition screening examination were not included [26, 27]. However, this group involved a small number of patients (*n*=31) in the second study only (Biese 2018) [26, 27]. More details concerning the risks of bias are presented in Additional Table 1.

### Main results: effect of TFU on health-related and patient-oriented outcomes

Both studies did not find a statistically significant effect of TFU in reduction of ED return visits, hospitalization, or death 30 or 35 days after ED discharge (Table 2) [26, 27].

In one study (Biese et al. 2014), patients in the TFU group had significantly more often a physician appointment scheduled within 5 days than patients in the placebo and the control group. However, the authors reported that for a minority of TFU group patients, the calling nurse helped to schedule appointments, which may have contributed to a shorter follow-up time [26]. In the other study (Biese et al. 2018), the authors found no benefit of the intervention on the number of scheduled physician follow-up appointments within 30 days after discharge from the ED. [27] Both studies did not report whether patients actually showed up on the planned appointments.

No significant differences between the groups were found in obtaining prescribed medication and in knowledge of name, dosage, or indication of the prescribed medication [26, 27].

### Feasibility in daily ED practice

In the included studies, eligible patients were approached for participation by telephone. In the Biese et al. 2014 study, all 178 eligible patients were reached, but in the Biese et al. 2018 study, 2712 (42%) of the 6463 eligible patients could not be reached and hence could not be approached for participation. During follow-up, the included patients were well accessible by telephone in both studies: ≥89% of the included patients was reached. Of the eligible patients who were reached and approached for participation, 10% declined to participate in the Biese et al. 2014 study, whereas in the Biese et al. 2018 study, 45% declined [26, 27]. In Biese’s 2014 study, patients were only enrolled after visits to the ED on Sunday, Monday, and Tuesday and not more than nine patients per day, to facilitate follow-up calls during the week, because they did not have enough staff to make calls during the weekend [26]. In Biese’s 2018 study, there were no restrictions for inclusion concerning the day and time of the ED visit and the number of inclusions per day [27].

## Discussion

Only two controlled studies, both RCTs, met the inclusion criteria for this review. Both studies reported no effect of a TFU call from a nurse for older patients, discharged home from the ED on hospital admission or ED return visit rates within 30 or 35 days after the index ED visit. However, only the Biese et al. 2018 study was powered to find a significant difference on this outcome [27]. The Biese et al. 2014 study reported that patients in the TFU group had significantly more often a physician appointment scheduled within 5 days than patients in the placebo and the control group. This effect was not found in the other included study, examining differences in scheduled physician appointments within 30 days. TFU was not shown to be helpful in obtaining prescribed medications or knowledge of name, dosage, and indication of prescribed medications.

Although patients who were included in the studies were well accessible by telephone for follow-up calls, many eligible patients were not reached and hence could not be approached for participation. Moreover, a substantial number of eligible patients refused to participate. This questions the feasibility of the intervention in daily practice.

The findings of the studies included in this systematic review are in accordance with other systematic reviews that examined the effects of TFU after hospital admission in (adult) patients of all ages. Crocker et al. evaluated the impact of TFU, performed by primary care personnel, after hospital admission on ED visit and hospital readmission rates in adults of all ages and did not found TFU to be beneficial [20]. Authors of a 2006 Cochrane review and a review of Bahr et al. found inconclusive evidence about the effects of TFU after hospital discharge. In the included studies, TFU was performed in a large variety of ways and by different kinds of health care professionals in different patient populations. Most studies were of low methodological quality, and many different outcomes were measured, ranging from outcomes related to health services utilization to physical and psychosocial health outcomes. Effects were not constant across the included studies and overall, and the evidence was inconclusive [13, 21]. In 2019 Nasser et al. published a review evaluating the effect of TFU on compliance with follow-up and discharge instructions in older patients, discharged home from the ED. It was concluded that TFU can identify non-compliance with discharge instructions, but evidence to improve compliance was not found [22].

Some previously published uncontrolled studies reported that TFU after ED discharge was feasible as only few patients declined participation or were not reached [17, 28]. The patients in the included studies in our review were also well accessible by phone for follow-up. However, this may reflect participation bias, as in one of the studies many eligible patients were not reached by phone and therefore could not be approached for inclusion. These may well have been patients with physical or other impairments who were unable to answer the telephone, but could have benefited from TFU [17]. Problems concerning telephone accessibility of patients are also mentioned in other studies [14, 21, 29]. Many studies report the lack of a correct phone number, which could be addressed by verifying the patient’s telephone number at discharge. The telephone number of a caregiver or family member can also be asked in case the patient cannot be reached for TFU. It is probable that for many older patients, involvement of family members or other caregivers in TFU increases accessibility and improves discharge plan adherence and other postdischarge outcomes [29, 30]. A substantial number of eligible patients refused to participate. This was also reported in a study, investigating the effect of telephone support calls by volunteers on feelings of loneliness and depression by older patients, discharged home from the ED. [31] Patients may have refused participation, because they did not want to be involved in a study, but they may also judge TFU as unnecessary interference. Although less time-consuming than other transitional care programs, TFU still requires sufficient staff to approach all eligible patients [21]. This is illustrated in the Biese et al. 2014 study, enrolling patients only on specific weekdays and up to a maximum of nine per day, because they did not have enough staff to perform more telephone calls [26]. Not including patients on other weekdays may undoubtedly have led to missing eligible patients who presented outside this inclusion window. The substantial number of eligible patients that was not reached or refused participation underlines the efforts that are needed to make FTU feasible in daily practice [26, 27, 31].

The studies included in this review investigated the effect of TFU on health services utilization and understanding of and compliance with discharge instructions. The effects of TFU on other, more difficult to measure outcomes, such as psychosocial health outcomes, were not measured. A systematic review investigating older patients’ expectations of emergency care reported that insufficient or poorly understood explanations about diagnosis or discharge instructions were associated with less satisfaction with care [32]. It may be that with TFU ED staff could meet these expectations by providing additional explanations and care. Besides that, TFU can be regarded as a socially complex intervention, characterized by difficult to define and to standardize interactions and by various contextual factors, which may mask potential effects. To support this idea, the Dutch Patients and Costumers Federation stated that TFU deserved a place in aftercare, despite the negative findings of the 2006 Cochrane review, because patients had indicated that they highly appreciated the call [13]. In accordance with this, some studies suggest that several older patients are in need of social and emotional support following an ED visit and that (repetitive) TFU could provide for this [28, 31]. It would be worth exploring in future research how care transition interventions after an ED visit affect perceived emotional and social support and specific needs and barriers that older ED users experience [30].

The limited number of controlled studies concerning this subject is remarkable, given the increasing number of proactive care programs for older patients in many EDs [27]. Apart from the two studies that met the inclusion criteria for this review, we found one more suitable study. This cohort study with pre-post design, published in Dutch in a non-peer reviewed journal, also reported no effect of TFU on hospital admission or ED return visit rate within 30 days after discharge from a general hospital ED. [33] The small number of available studies, all showing no benefit of the intervention may underline the absence of effect of TFU on health-related outcomes. More controlled intervention studies are needed to investigate the effect of TFU in older ED patients. Future studies should best focus the intervention on individuals at highest risk of hospital use, such as those with functional or cognitive impairments, with mental health conditions, limited social support, or with complex medical regimens, to determine whether there are different effects of TFU in these populations [1, 30, 34]. Interesting outcome measures, in addition to health service utilization, would be functional decline, perceived social, and emotional support and feelings of anxiety or depression. Failure to reach eligible patients could be addressed by appointing sufficient staff members to perform the intervention, by verifying the patient’s telephone number at discharge and by involving the patients’ caregivers. It would also be interesting to investigate the effects and feasibility of TFU performed by other personnel than ED staff, e.g., primary care personnel or nurses from a commercial call center.

### Strengths and limitations

#### Strengths

In this systematic review, only quantitative, controlled studies were included. Both included studies were RCTs and serious efforts had been made to limit the risks of bias. The risk of missing relevant publications was minimized by searching multiple databases and trial websites and by assessing citations and full-text articles for eligibility by two reviewers.

#### Limitations

The two RCTs included in this review were conducted in the same tertiary ED in the USA. This may limit generalizability of the study results to other countries. However, a Dutch study did not show a beneficial effect of TFU either [33]. Only one of the studies was of sufficient sample size to detect a significant effect of TFU on hospitalization and ED return visits. This study compared TFU with a telephone satisfaction survey call, but not with no telephone call. In future research, it would be worth comparing the outcomes of patients receiving TFU with those of patients who do not receive any telephone intervention. Patients or their caregivers or spouses who did not pass the mental cognition screening examination were excluded from both studies. Although cognitively impaired, these individuals might have benefited from a telephone intervention. However, the number of patients excluded for this reason was limited. Due to the small number of included studies, the heterogeneity of the study methods and the negative results, a quantitative analysis of the studies, including assessment of heterogeneity and publication bias by creating a funnel plot, was considered not to be of added value. Therefore, we used a qualitative approach to synthesize the literature.

## Conclusions

Telephone follow-up is considered to be a low-cost intervention, that probably allows the opportunity to detect problems and complications, clarify discharge instructions, and initiate other forms of aftercare for older adults discharged home from the ED. However, our systematic review of two published randomized controlled studies found no demonstrable effect of TFU for older adults, discharged from the ED on health service utilization and understanding of and compliance with discharge instructions. Furthermore, feasibility of the intervention appeared to be low. Considering the limited number of high-quality studies on this topic, more research is needed to explore whether TFU is an effective and feasible intervention to reduce hospitalization and ED return visit rates or to improve older patients’ discharge plan adherence after an ED visit. In future studies, it is important to also investigate whether TFU promotes psychosocial wellbeing in older patients after ED discharge.

## Supplementary Information

**Additional file 1.** Search strategy.

**Additional file 2.** Overview of websites that were searched on 1 December 2019 to identify eligible articles and studies.

**Additional file 3.** Data extraction template.

**Additional file 4.** Additional table 1: Risk of bias of the included studies on seven domains.

## Data Availability

Full details of the search strategy are available in appendix A and a list of searched clinical trial websites is presented in appendix B. More details concerning the study selection process and completed data extraction sheets of the included publications are available from the corresponding author on reasonable request.

## Abbreviations

ED: Emergency Department
TFU: Telephone follow-up
PRISMA: Preferred Reporting Items for Systematic Reviews and Meta-Analyse
RCT: Randomized Controlled Trial
*n*: number
et al.: et alii, meaning “and others”
