# Supplementary material for: The effect of a telephone follow-up call for older patients, discharged home from the emergency department on health-related outcomes: a systematic review of controlled studies
Source: Int J Emerg Med. 2021 Feb 18;14:13. doi: 10.1186/s12245-021-00336-x (PMC7893958; doi:10.1186/s12245-021-00336-x)
Supplement: Supplementary file 1 — Additional file 1. Search strategy. [file 12245_2021_336_MOESM1_ESM.docx]

**Additional file 1:** search strategy

| **Date** | **Database** | **Strategy** | **Number of references** |
| --- | --- | --- | --- |
| 09-12-2019 | PubMed ([www.pubmed.gov](http://www.pubmed.gov)) | 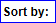  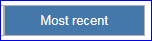  ("Aged"[Mesh] OR aged*[tiab] OR aging*[tiab] OR ageing*[tiab] OR elder*[tiab] OR geriatr*[tiab] OR geront*[tiab] OR frail*[tiab] OR octogenarian*[tiab] OR octo-genarian*[tiab] OR nonagenarian*[tiab] OR nona-genarian*[tiab] OR non-agenarian*[tiab] OR centenarian*[tiab]) AND ("Emergency Medical Services"[Mesh] OR "Emergency Services, Psychiatric"[Mesh] OR "Emergency Treatment"[Mesh] OR "Emergency Nursing"[Mesh] OR "Emergency Medicine"[Mesh] OR emergenc*[tiab] OR emer-genc*[tiab] OR "ed"[tiab] OR "eds"[tiab] OR ed's*[tiab] OR "er"[tiab] OR "ers"[tiab] OR er's*[tiab] OR accident department*[tiab] OR "accident dept"[tiab] OR (trauma*[ti] AND (center*[ti] OR centre*[ti])) OR trauma center*[tiab] OR trauma centre*[tiab] OR (trauma*[ti] AND hospital*[ti]) OR trauma hospital*[tiab] OR (acute*[ti] AND (service*[ti] OR care*[ti] OR centre*[ti] OR center*[ti])) OR acute service*[tiab] OR acute care*[tiab] OR acute center*[tiab] OR acute centre*[tiab] OR (urgen*[ti] AND (service*[ti] OR care*[ti] OR centre*[ti] OR center*[ti])) OR urgency service*[tiab] OR urgent service*[tiab] OR urgent care*[tiab] OR urgent center*[tiab] OR acute centre*[tiab] OR urgent-centre*[tiab]) AND (("Aftercare"[Mesh] AND "Telephone"[Mesh]) OR post-discharge follow-up*[tiab] OR postdischarge follow-up*[tiab] OR post-discharge-followup*[tiab] OR postdischarge followup*[tiab] OR (interven*[ti] AND (phone*[ti] OR telephon*[ti])) OR ((phone*[ti] OR telephon*[ti]) AND (postdischarge*[ti] OR discharge*[ti] OR follow-up*[ti] OR followup*[ti])) OR postdischarge phon*[tiab] OR post-discharge-phon*[tiab] OR postdischarge telephon*[tiab] OR post-discharge telephon*[tiab] OR discharge-phon*[tiab] OR discharge telephon*[tiab] OR phone follow-up*[tiab] OR phone-followup*[tiab] OR telephone follow-up*[tiab] OR telephone followup*[tiab] OR follow-up phon*[tiab] OR followup-phon*[tiab] OR follow-up telephon*[tiab] OR followup telephon*[tiab]) AND ("Clinical Trial" [Publication Type] OR "Comparative Study" [Publication Type] OR "Evaluation Studies" [Publication Type] OR "Cross-Over Studies"[Mesh] OR "Multicenter Study" [Publication Type] OR "Random Allocation"[Mesh] OR "Double-Blind Method"[Mesh] OR "Single-Blind Method"[Mesh] OR "Placebos"[Mesh] OR "Research Design"[Mesh:NoExp] OR "trial"[tiab] OR trial'*[tiab] OR random*[tiab] OR placebo*[tiab] OR sham*[tiab] OR comparison*[tiab] OR controlled-clinical-trial*[tiab] OR controlled-clinical-stud*[tiab] OR crossover*[tiab] OR cross-over*[tiab] OR double-blind*[tiab] OR doubleblind*[tiab] OR "group"[tiab] OR group'*[tiab] OR groups*[tiab] OR "control"[tiab] OR control'*[tiab] OR "controls"[tiab] OR controls'*[tiab] OR controll*[tiab] OR controlgroup*[tiab] OR volunteer*[tiab] OR ((singl*[tiab] OR doubl*[tiab] OR trebl*[tiab] OR tripl*[tiab]) AND (mask*[tiab] OR blind*[tiab])) OR latin-square*[tiab] OR multicenter*[tiab] OR multi-center*[tiab] OR multicentre*[tiab] OR multi-centre*[tiab] OR 4-arm*[tiab] OR four-arm*[tiab]) | 252 |
| 09-12-2019 | Embase  - OVID-version  - 1974 to 2019 december 06.  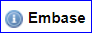  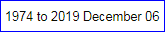 | 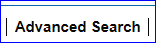  (exp aged/ OR aged*.ti,ab,kw. OR aging*.ti,ab,kw. OR ageing*.ti,ab,kw. OR elder*.ti,ab,kw. OR geriatr*.ti,ab,kw. OR geront*.ti,ab,kw. OR frail*.ti,ab,kw. OR octogenarian*.ti,ab,kw. OR octo-genarian*.ti,ab,kw. OR nonagenarian*.ti,ab,kw. OR nona-genarian*.ti,ab,kw. OR non-agenarian*.ti,ab,kw. OR centenarian*.ti,ab,kw.) AND (exp emergency health service/ OR exp emergency medical dispatch/ OR exp hospital emergency service/ OR exp psychiatric emergency service/ OR exp emergency treatment/ OR exp emergency nursing/ OR exp emergency medicine/ OR emergenc*.ti,ab,kw. OR emer-genc*.ti,ab,kw. OR "ed".ti,ab,kw. OR "eds".ti,ab,kw. OR ed's*.ti,ab,kw. OR "er".ti,ab,kw. OR "ers".ti,ab,kw. OR er's*.ti,ab,kw. OR accident department*.ti,ab,kw. OR "accident dept".ti,ab,kw. OR (trauma*.ti. AND (center*.ti. OR centre*.ti.)) OR trauma center*.ti,ab,kw. OR trauma centre*.ti,ab,kw. OR (trauma*.ti. AND hospital*.ti.) OR trauma hospital*.ti,ab,kw. OR (acute*.ti. AND (service*.ti. OR care*.ti. OR centre*.ti. OR center*.ti.)) OR acute service*.ti,ab,kw. OR acute care*.ti,ab,kw. OR acute center*.ti,ab,kw. OR acute centre*.ti,ab,kw. OR (urgen*.ti. AND (service*.ti. OR care*.ti. OR centre*.ti. OR center*.ti.)) OR urgency service*.ti,ab,kw. OR urgent service*.ti,ab,kw. OR urgent care*.ti,ab,kw. OR urgent center*.ti,ab,kw. OR acute centre*.ti,ab,kw. OR urgent-centre*.ti,ab,kw.) AND (((exp rehabilitation/ OR aftercare/) AND exp telephone/) OR post-discharge follow-up*.ti,ab,kw. OR postdischarge follow-up*.ti,ab,kw. OR post-discharge-followup*.ti,ab,kw. OR postdischarge followup*.ti,ab,kw. OR (interven*.ti. AND (phone*.ti. OR telephon*.ti.)) OR ((phone*.ti. OR telephon*.ti.) AND (postdischarge*.ti. OR discharge*.ti. OR follow-up*.ti. OR followup*.ti.)) OR postdischarge phon*.ti,ab,kw. OR post-discharge-phon*.ti,ab,kw. OR postdischarge telephon*.ti,ab,kw. OR post-discharge telephon*.ti,ab,kw. OR discharge-phon*.ti,ab,kw. OR discharge telephon*.ti,ab,kw. OR phone follow-up*.ti,ab,kw. OR phone-followup*.ti,ab,kw. OR telephone follow-up*.ti,ab,kw. OR telephone followup*.ti,ab,kw. OR follow-up phon*.ti,ab,kw. OR followup-phon*.ti,ab,kw. OR follow-up telephon*.ti,ab,kw. OR followup telephon*.ti,ab,kw.) AND (exp clinical trial/ OR exp comparative study/ OR exp evaluation study/ OR exp crossover procedure/ OR exp multicenter study/ OR exp randomization/ OR exp double blind procedure/ OR exp single blind procedure/ OR exp placebo/ OR "trial".ti,ab,kw. OR trial'*.ti,ab,kw. OR random*.ti,ab,kw. OR placebo*.ti,ab,kw. OR sham*.ti,ab,kw. OR comparison*.ti,ab,kw. OR controlled-clinical-trial*.ti,ab,kw. OR controlled-clinical-stud*.ti,ab,kw. OR crossover*.ti,ab,kw. OR cross-over*.ti,ab,kw. OR double-blind*.ti,ab,kw. OR doubleblind*.ti,ab,kw. OR "group".ti,ab,kw. OR group'*.ti,ab,kw. OR groups*.ti,ab,kw. OR "control".ti,ab,kw. OR control'*.ti,ab,kw. OR "controls".ti,ab,kw. OR controls'*.ti,ab,kw. OR controll*.ti,ab,kw. OR controlgroup*.ti,ab,kw. OR volunteer*.ti,ab,kw. OR ((singl*.ti,ab,kw. OR doubl*.ti,ab,kw. OR trebl*.ti,ab,kw. OR tripl*.ti,ab,kw.) AND (mask*.ti,ab,kw. OR blind*.ti,ab,kw.)) OR latin-square*.ti,ab,kw. OR multicenter*.ti,ab,kw. OR multi-center*.ti,ab,kw. OR multicentre*.ti,ab,kw. OR multi-centre*.ti,ab,kw. OR 4-arm*.ti,ab,kw. OR four-arm*.ti,ab,kw.) | 297 |
| 09-12-2019 | Cochrane Library | 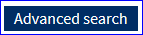  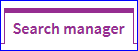  Four *separated* searches, combined *afterwards*:  (aged* OR aging* OR ageing* OR elder* OR geriatr* OR geront* OR frail* OR octogenarian* OR (octo NEXT genarian*) OR nonagenarian* OR (nona NEXT genarian*) OR (non NEXT agenarian*) OR centenarian*):ti,ab,kw  AND  (emergenc* OR (emer NEXT genc*) OR "ed" OR "eds" OR "er" OR "ers" OR (accident NEXT department*) OR (accident NEXT dept) OR (trauma NEXT center*) OR (trauma NEXT centre*) OR (trauma NEXT hospital*) OR (acute NEXT service*) OR (acute NEXT care*) OR (acute NEXT center*) OR (acute NEXT centre*) OR (urgency NEXT service*) OR (urgent NEXT service*) OR (urgent NEXT care*) OR (urgent NEXT center*) OR (acute NEXT centre*) OR (urgent NEXT centre*)):ti,ab,kw OR ((trauma* AND (center* OR centre*)) OR (trauma* AND hospital*) OR (acute* AND (service* OR care* OR centre* OR center*)) OR (urgen* AND (service* OR care* OR centre* OR center*))):ti  AND  ((post NEXT discharge NEXT follow NEXT up*) OR (postdischarge NEXT follow NEXT up*) OR (post NEXT discharge NEXT followup*) OR (postdischarge NEXT followup*) OR (postdischarge NEXT phon*) OR (post NEXT discharge NEXT phon*) OR (postdischarge NEXT telephon*) OR (post NEXT discharge NEXT telephon*) OR (discharge NEXT phon*) OR (discharge NEXT telephon*) OR (phone NEXT follow NEXT up*) OR (phone NEXT followup*) OR (telephone NEXT follow NEXT up*) OR (telephone NEXT followup*) OR (follow NEXT up NEXT phon*) OR (followup NEXT phon*) OR (follow NEXT up NEXT telephon*) OR (followup NEXT telephon*)):ti,ab,kw OR ((interven* AND (phone* OR telephon*)) OR ((phone* OR telephon*) AND (postdischarge* OR discharge* OR (follow NEXT up*) OR followup*))):ti  AND  ("trial" OR trial'* OR random* OR placebo* OR sham* OR comparison* OR (controlled NEXT clinical NEXT trial*) OR (controlled NEXT clinical NEXT stud*) OR crossover* OR (cross NEXT over*) OR (double NEXT blind*) OR doubleblind* OR "group" OR group'* OR groups* OR "control" OR control'* OR "controls" OR controls'* OR controll* OR controlgroup* OR volunteer* OR ((singl* OR doubl* OR trebl* OR tripl*) AND (mask* OR blind*)) OR (latin NEXT square*) OR multicenter* OR (multi NEXT center*) OR multicentre* OR (multi NEXT centre*) OR (4 NEXT arm*) OR (four NEXT arm*)):ti,ab,kw | 199 |
