# Supplementary material for: The effect of a telephone follow-up call for older patients, discharged home from the emergency department on health-related outcomes: a systematic review of controlled studies
Source: Int J Emerg Med. 2021 Feb 18;14:13. doi: 10.1186/s12245-021-00336-x (PMC7893958; doi:10.1186/s12245-021-00336-x)
Supplement: Supplementary file 2 — Additional file 2. Overview of websites that were searched on 1 December 2019 to identify eligible articles and studies. [file 12245_2021_336_MOESM2_ESM.docx]

**Additional file 2:**

Overview of websites that were searched on 1 December 2019 to identify eligible articles and studies

Netherlands Trial Register: [www.trialregister.nl](http://www.trialregister.nl)

ClinicalTrials.gov: https://ClinicalTrials.gov/

Australian Clinical Trials: https://www.australianclinicaltrials.gov.au/

Australian New Zealand Clinical Trials Registry: http://www.anzctr.org.au/

World Health Organization's International Clinical Trials Registry Platform: http://apps.who.int/trialsearch/

EU Clinical Trials Register: https://www.clinicaltrialsregister.eu/

OpenGrey: http://www.opengrey.eu/

Google Scholar
