# Supplementary material for: The effect of a telephone follow-up call for older patients, discharged home from the emergency department on health-related outcomes: a systematic review of controlled studies
Source: Int J Emerg Med. 2021 Feb 18;14:13. doi: 10.1186/s12245-021-00336-x (PMC7893958; doi:10.1186/s12245-021-00336-x)
Supplement: Supplementary file 3 — Additional file 3. Data extraction template. [file 12245_2021_336_MOESM3_ESM.doc]

**Additional file 3:** Data extraction template

**Form version/date** *(eg. Version 1.4, 5 August 2019)*

# Review Title

# Study ID *(Surname and Year)*

# Name of review author completing this form

## Date form completed

# Name of review author checking the data extracted to this form

**Other information and notes**

| **Author contact details for study** |  |
| --- | --- |
| **Further information required** |  |
| **Correspondence with authors successful or not; what information was received and when** |  |
| **Will any additional unpublished data supplied by the authors be included in the review?**  **If so, note that the study will include unpublished data** |  |
| **Notes** *(Unpublished – for own use)*  *eg. references to be followed up, source of information especially if multiple reports of same trial, or unpublished data/personal communication included.* | |

**Section 2: Methods of the study**

*Details of Study (to be reported in the Characteristics of Included Studies tables)*

Aim of study *(As stated in the trial report/s. What was the trial designed to assess?)*

Study design

Number of arms or groups (including control groups); briefly describe each

Consumer involvement *(eg. In design of study and/or intervention; in delivery of intervention; in evaluation of intervention; in interpretation of study findings)*

Funding source (also include any details about possible or explicit conflicts of interest)

Informed consent obtained? *(Yes/No/Unclear)*

Ethical approval *(Yes/No/Unclear)*

**Section 3: Risk of Bias assessment**

**This has been adapted directly from Cochrane Handbook: The Cochrane Collaboration’s tool for assessing risk of bias**

| **Domain** | **Review authors’ judgement** | **Instructions** | **Notes on rating** |
| --- | --- | --- | --- |
| **Random sequence generation^1^** | ***High risk***  ***Unclear***  ***Low risk*** | Describe the method used to generate the allocation sequence in sufficient detail to allow an assessment of whether it should produce comparable groups. | Quasi-RCTs and Controlled Before and After (CBA) studies must be rated as ‘High risk’ for random sequence generation as the methods were not, by definition, truly random.  If you are including only RCTs in your review, papers marked ‘High risk’ should be excluded as they are not truly randomised.  Note that to exclude a study on this basis there must be agreement on this decision by at least two authors. |
| **Allocation concealment** | ***High risk***  ***Unclear***  ***Low risk*** | Describe the method used to conceal the allocation sequence in sufficient detail to determine whether intervention allocations could have been foreseen in advance of, or during, enrolment. | Quasi-RCTs are likely to be rated ‘High risk' but there may be exceptions.  CBA Studies should be rated ‘High risk. |
| **Blinding of participants and personnel**  *Assessments should be made for each main outcome (or class of outcomes)* | ***High risk***  ***Unclear***  ***Low risk*** | Describe all measures used, if any, to blind study participants and personnel from knowledge of which intervention a participant received.  Note that the impact of performance bias must be considered and reported even if blinding of participants and/or personnel is not possible for the type of intervention being evaluated^2,3^ | Consider:   1. Did the study attempt to blind the participants and/or personnel so that they did not know who received the intervention? Note that it may be possible to blind one but not the other (*eg participants but not personnel, or vice versa*) 2. Were the measures that the study took to blind participants and/or personnel to study groups effective (or not)?   These points will help to make the decision about whether the study is likely to be affected by performance bias (high, unclear, or low risk).  Even in studies of informational or educational interventions it may be possible (though difficult) to effectively blind participants and/or personnel to intervention status (*eg measures such as a 'placebo' video, control information brochure, blank instructional booklet*).  Please note that when making sense of the risk of bias ratings, you will need to consider the effects of blinding and incomplete outcome data by outcome, not just by study. |
| **Blinding of outcome assessment**  *Assessments should be made for each main outcome (or class of outcomes)* | ***High risk***  ***Unclear***  ***Low risk*** | 1. Describe all measures used, if any, to blind outcome assessors from knowledge of which intervention a participant received.  2. Provide any information relating to whether the intended blinding was effective. Blinding of outcome assessment can be feasible even if blinding of participants and personnel is not. | The implications of whether outcome assessment was blinded, and how effectively, may differ across outcomes. Blinding of outcome assessment should therefore be considered separately for each outcome.^3^  Outcomes may be assessed using subjective or objective measures, and by self-reported or other means. They may be assessed by research personnel or by participants.  To deal with this complexity, the following points are suggested as a guide:  For **personnel-measured** outcomes:  *eg case notes, observed medicine taking, rate of participation*   - - Participants blinded     - Personnel blinded: LOW risk     - Personnel not blinded: HIGH risk   - Participants not blinded     - Personnel blinded: UNCLEAR risk     - Personnel not blinded: HIGH risk   For **self-reported outcomes:**  *eg knowledge, self-reported compliance, anxiety*   - - Participants blinded     - Personnel blinded: LOW risk     - Personnel not blinded or unclear whether blinded: UNCLEAR risk   - Participants not blinded     - Personnel blinded or unclear whether blinded: UNCLEAR risk     - Personnel not blinded: HIGH risk |
| **Incomplete outcome data**  *Assessments should be made for each main outcome (or class of outcomes)* | ***High risk***  ***Unclear***  ***Low risk*** | Describe the completeness of outcome data for each main outcome, including attrition (loss to follow up, withdrawn) and exclusions from the analysis. Note that the participant numbers and reasons reported in the ‘Participants’ section of this form (below) should be used as a basis for making these decisions.  State whether attrition and exclusions were reported, the numbers in each intervention group (compared with total randomized participants), reasons for attrition/exclusions where reported, and any re-inclusions in analyses performed by the review authors. | The following ratings are suggested as a guide for rating this item:  **High risk**   - Reasons for missing data are related to the outcome, and there is imbalance in numbers or reasons for missing data across study groups (*eg more people dropped out of the intervention than control group because of adverse events of a study medication*). - The proportion of data missing or plausible effect size is large enough to have a clinically relevant effect. - Analysis was not performed on an ‘intention to treat’ basis (where people are analysed in the groups to which they were randomly assigned, irrespective of what happened during the study). - Imputation (entering substitute data to take the place of missing data) was done inappropriately.   **Unclear risk**   - The data is poorly reported - it is not clear how many participants/ data were lost from the study groups, and/or what the reasons for missing data were.   **Low risk**   - No data is missing. - Reasons for missing data are not related to the outcome. - Missing data is balanced across the study groups, and reasons for missing data are similar across groups. - The proportion of data missing or plausible effect size is not large enough to have a clinically relevant effect.   The impact of missing data must be assessed for each outcome (or group of outcomes), as it may vary, and must also be considered at different time points if data was collected at different times.  Assessing the completeness of outcome data must take into account:   1. How much data is missing from each group? 2. Why is it missing? 3. How was the data analysed? 4. No simple rule applies across the board; although the overall proportion of missing data is one thing to consider (*eg 50% of data missing would be more of a concern than 5%*). However, a judgement about attrition bias also relies on an assessment of whether enough data is missing that it could meaningfully affect the results. Assessing this means considering:  - For dichotomous data: is the outcome rare or more common? If rare, only a few missing data could change the conclusions, whereas if the outcome is more common much more data could be missing before the conclusions would be altered. - For continuous data: could the values for the missing participants be extremely different to the calcuated mean for the sample available? If the missing values could not be very different to the mean value, it would take a lot of missing data to alter the mean. On the other hand, if the missing values could be very different to the estimated mean value, fewer missing data could produce a different mean.  1. Reasons for missing data must also be considered. If the reason is not related to the outcome (eg people moved house and could no longer participate), this is described as data missing at random and is unlikely to systematically influence (bias) the results. If the reason for missing data is related to the outcome however, and this is different across study groups (*eg more people dropped out of the intervention than control group because of adverse events of a study medication*), this can introduce bias. 2. Different re-analysis techniques may disrupt the randomisation set up for an RCT and so should be looked at carefully when assessing this risk of bias item. Refer to online training materials and Handbook.   Please note that when making sense of the risk of bias ratings, you will need to consider the effects of blinding and incomplete outcome data by outcome, not just by study. |
| **Selective reporting** | ***High risk***  ***Unclear***  ***Low risk*** | State how the possibility of selective outcome reporting was examined by the review authors, and what was found. | The following ratings are suggested as a guide:  **High risk:**   - If a protocol for the study is available, and outcomes identified in the protocol are not reported by the study; and/or - Outcomes reported in the methods section are not reported as planned (ie as results for the study); and/or - Expected outcomes are reported but done in such a way that they cannot be included in the review’s analyses (*eg the study reports a result as ‘statistically significant’; but does not provide the specific numerical or other data that could be included in the analysis of that outcome*).   **Unclear risk:**   - If no protocol for the study is available (and all expected outcomes reported in the methods are reported as planned)   **Low risk:**   - A protocol for the study is available and all expected outcomes are identified and reported as planned by the study. |
| **Other sources of bias** | ***Note: all answers should follow the format:***  ***High risk***  ***Unclear***  ***Low risk*** | State any important concerns about bias not addressed in the other domains in the tool. | If particular questions/entries were pre-specified in the review’s protocol, responses should be provided for each question/entry.  Note that any other sources of bias identified here must have the potential to introduce systematic errors in the results of the study (not involve other aspects of the study that should be reported elsewhere in the review).  Assessing other sources of bias is not essential but should be guided by the study designs included in the review.  Do not assess in this domain aspects of conduct of the study, such as those:   - associated with the ‘quality’ of a study *eg ethical criteria – such as whether the study obtained ethics approval;* - related to precision of the study *eg use of a power calculation* - linked to reporting standards or - related to validity and/or reliability of outcome measures   These aspects of the study can be collected and reported in the ‘Characteristics of included studies’ table. |

**1** Please note that contact with authors of an included study may mean that some decisions need to be revised. For example, if information from study authors confirms that the allocation method was not truly randomised, even if the study report describes the study as an RCT, (and only RCTs were eligible for inclusion in the review), the study would then need to be excluded from the review.

**2** For example: if participants and personnel cannot be blinded effectively to the intervention, this item would be rated as at high risk of bias for performance bias, with a reason for this decision reported as (for example) ‘Participants and personnel were not able to be blinded to intervention’ in the risk of bias tables.

**3** For example, objective outcome measures (eg chart review, electronically recorded medicine taking, mortality) might be less affected by a lack of blinding than the potential effect of unblinded outcome assessment on subjective outcomes (eg pain, self-reported adherence, quality of life). Similarly, for blinding of participants and personnel the risk of bias may be high for some outcomes if unblinded (eg behavioural, socially desirable or some self-reported outcomes) but less likely to affect others such as mortality.

**Section 4: Study characteristics - Participants**

Description *(eg. Patients/consumers; carers; parents of patients/consumers; health professionals; well people in the community)*

Geographic location *(eg. City/State/Country)*

Setting (*eg. Community, home, primary health centre, acute care hospital, extended care facility)*

Methods of recruitment of participants *(How were potential participants approached and invited to participate?)*

Inclusion/exclusion criteria for participation in study

Number*s* involved:

| **Study numbers** | **Number** |
| --- | --- |
| Eligible for inclusion |  |
| Excluded |  |
| Refused to take part |  |
| Randomised to intervention group(s) |  |
| Randomised to control group |  |
| Excluded post randomisation (for each group; with reasons if relevant) |  |
| Withdrawn (for each group; with reasons if relevant) |  |
| Lost to follow up (for each group; with reasons) | Intervention group (with reasons) |
|  | Control group (with reasons) |
| Included in the analysis (for each group, for each outcome) | Outcome 1  Intervention  Control |
|  | Outcome 2  Intervention  Control |
|  | Outcome 3  Intervention  Control |
|  | Outcome 4  Intervention  Control |
|  | Outcome 5  Intervention  Control |

**Section 5: Study characteristics - Interventions**

Data should be extracted for each relevant (included) intervention arm, as well as the control arm. Information on any co-interventions (if applicable) should also be recorded.

| **Item** | **Explanation, notes** | **Intervention** | **Control or usual care** |
| --- | --- | --- | --- |
| **1 Intervention name** | Include a brief name or phrase that describes the intervention  (*including definition of any acronyms or abbreviations*) |  |  |
| **2 Aims and rationale ('why?')** | Aim(s) of intervention  *(as stated in the trial report/s. What was the problem that this intervention was designed to address?)* |  |  |
| **3 What was done?** | Materials:  Describe the content, format(s) or media, source of materials (if possible, where they can be accessed), and any other information relevant to the physical or information materials provided to participants or in training providers of the intervention.  Procedures:  Describe each of the processes used in delivering the intervention *(eg education, telephone follow-up, case management)*  *Note that some complex interventions require additional support activities to be implemented, and if so details of these should also be reported.*  *Note also that some complex interventions require sequencing of activities, whereas for others the order of delivery is less critical.*  Mode of delivery:  Describe the mode of delivery of the intervention, such as whether it was delivered face-to-face (*eg in patient consultation, educational session, training*) or at a distance *(eg via phone, internet, mail);* and whether the delivery was to individuals or groups of participants.  Co-interventions:  Describe the delivery of any co-interventions  (*Co-interventions may be separate to the intervention of interest, or they may be other similar elements in a suite of interventions which have a common purpose*). |  |  |
| **4 Who delivered the intervention?** | Describe who was involved in delivery of each component of the intervention and/or each different intervention provider.  ‘Intervention provider’ could for example be taken to mean a health professional or it could mean a consumer peer advocate.  Include description of any specific training given to providers to deliver the intervention, numbers of providers, professional background, specific pre-existing skills or experience required, quality of any specific training received to deliver the intervention, and any measures of competence or consistency in delivering the intervention recorded before or during the study. |  |  |
| **6 Where was the intervention provided?** | Describe the features of the setting (location) that might be relevant to intervention delivery  *(eg country, type of clinic, primary or hospital care).*  If the location varied this should be described, with relevant features that might affect the intervention delivery; as should any requisite features of the location that might impact on intervention delivery or feasibility  (*eg location close to participants' usual doctor, availability of equipment)* |  |  |
| **7 When and how often or how much of the intervention was provided?** | Describe how the intervention was delivered, such as stages, timing, frequency, number of sessions, intensity and duration of intervention delivery. |  |  |
| **8 Was the intervention tailored?** | If the intervention was meant to be tailored or personalised in the course of the study, describe the rationale for this and the major features of what was done - such as:   - how? - why? - when? and - what?   was done to tailor the intervention.  If particular decision rules were used to determine when or how to tailor the intervention details should be provided. |  |  |
| **9 Was the intervention modified or adapted?** | If the intervention was changed during the study, this should be described  (*eg unforseen modifications required, changes in study circumstances requiring modifications to the intervention).*  If such modifications happen, why, what, how and when the intervention was changed should be described. |  |  |
| **10 How well was the intervention delivered?** | Assessment of fidelity: if intervention fidelity was assessed, describe the extent to which the intervention was delivered as intended.  (*ie the amount or type of intervention planned for delivery might differ from what was actually delivered*)  If strategies to maintain intervention fidelity were planned before intervention delivery, or were used during the study, describe these, along with any materials or tools used. |  |  |

**Table is adapted from Hoffman et al (2014). Better reporting of interventions: template for intervention description and replication (TIDieR) checklist and guide. BMJ; 348:g1687.

**Section 6: Study characteristics - Outcomes and comparison groups**

**Please also note** that it may be useful to include a note about the direction of the effect alongside your extracted data. This may be helpful especially in cases where a number of different scales are used to report findings (across studies) and/or when sometimes an effect of an intervention is framed as a positive effect (eg increased symptom-free days) and as a negative effect (eg decrease in symptoms). This will help to ensure that there are no errors introduced once the extracted data is brought together across different studies (for a given outcome).

| **Primary outcomes** | | | |
| --- | --- | --- | --- |
| Outcome | Method of assessing outcome measures  *eg phone survey, questionnaire* | Method of follow-up for non-respondents | Timing of outcome assessment  *(including frequency, length of follow up)* |
|  |  |  |  |
|  |  |  |  |
|  |  |  |  |
|  |  |  |  |
|  |  |  |  |
|  |  |  |  |
|  |  |  |  |

| **Secondary outcomes** | | | |
| --- | --- | --- | --- |
| Outcome | Method of assessing outcome measures  *eg, phone survey, questionnaire* | Method of follow-up for non-respondents | Timing of outcome assessment  *(including frequency, length of follow up)* |
|  |  |  |  |
|  |  |  |  |
|  |  |  |  |
|  |  |  |  |
|  |  |  |  |
|  |  |  |  |
|  |  |  |  |

# Notes field

*For example:*

- *Contact with author (Yes (information obtained)/No) (SEE NOTE ON PAGE 1)*
- *Record if the study was translated from a language other than English.*
- *Record if the study was a duplicate publication.*

**Section 7: Data and results**

### All data are numbers (of patients/units), not percentages.

***Dichotomous outcomes***

| Outcome | Timing of outcome assessment (days/months) | Intervention group* | | Control group | | Notes |
| --- | --- | --- | --- | --- | --- | --- |
|  |  | Observed (n) | Total (N) | Observed (n) | Total (N) |  |
|  |  |  |  |  |  |  |
|  |  |  |  |  |  |  |
|  |  |  |  |  |  |  |
|  |  |  |  |  |  |  |
|  |  |  |  |  |  |  |
|  |  |  |  |  |  |  |

**Note: add additional columns if there is more than one intervention group, eg. Intervention Group A, Intervention Group B…*

# *Continuous outcomes*

| Outcome | Timing of outcome assessment (days/months) | Intervention group | | | Control group | | | Notes |
| --- | --- | --- | --- | --- | --- | --- | --- | --- |
|  |  | *Mean / Mean change | Standard deviation | N | *Mean / Mean change | Standard deviation | N |  |
|  |  |  |  |  |  |  |  |  |
|  |  |  |  |  |  |  |  |  |
|  |  |  |  |  |  |  |  |  |
|  |  |  |  |  |  |  |  |  |
|  |  |  |  |  |  |  |  |  |
|  |  |  |  |  |  |  |  |  |

**delete as appropriate*

***Other results or data:***

For example:

- additional data collected only for some participants that may be important for understanding the effects of the interventions (particularly if they relate to primary outcomes and/or adverse events)
- qualitative data that sits alongside the evaluation of effectiveness
- statements about the effects of interventions, reported without the numerical or supporting data (eg reported as 'knowledge was significantly higher in the intervention group'). Note that if this kind of data is reported in the review it must be clearly identified as such.
