# Supplementary material for: The effect of a telephone follow-up call for older patients, discharged home from the emergency department on health-related outcomes: a systematic review of controlled studies
Source: Int J Emerg Med. 2021 Feb 18;14:13. doi: 10.1186/s12245-021-00336-x (PMC7893958; doi:10.1186/s12245-021-00336-x)
Supplement: Supplementary file 4 — Additional file 4. Additional table 1: Risk of bias of the included studies on seven domains. [file 12245_2021_336_MOESM4_ESM.docx]

Additional table 1: Risk of bias of the included studies on seven domains.

| **Author, date,**  **country,**  **setting** | **Random sequence generation** | **Allocation concealment** | **Blinding of participants/ personnel** | **Blinding of outcome assessment** | **Incomplete outcome data** | **Selective reporting** | **Other bias** |
| --- | --- | --- | --- | --- | --- | --- | --- |
| Biese et al,  2014, USA, academic center ED | Blinded, block randomiza-  tion | Blinded, using marbles in a bag | Patients were blinded.  Nurse who did intervention was not blinded. Telephone calls were scripted. | Research assistants who did data collection phone calls were blinded for randomization, but might have known who was in the control group, as they had to perform a mental screening test only in control group patients, whereas other patients were tested earlier. | Incomplete data of 6 (4.5%) patients. 37 (23.6%) eligible patients were not included, due to refusal or not being reached.  Unclear whether patients were analyzed according to intention to treat. | Research protocol published in advance. Methods are followed and expected outcomes reported as planned. | Single center  Most outcome data were self-reported by patients.  Unknown how often the nurse helped patients making follow-up appointments.  Exclusion of potentially important individuals: patients not instructed to seek outpatient follow-up, patients visiting the ED in the weekend and patients and caregivers who did not pass the mental cognition screening examination. |
| Biese et al,  2018, USA, academic center ED | Randomiza-  tion with randomly generated block sizes of 4, 6 and 8. | Blinded, using a random sequence generator, imbedded in the computer program | Patients were blinded.  Nurses who did intervention were not blinded. Calls were scripted, recorded and reviewed to ensure adherence to the scripts. | Investigators were blinded for randomization. Unclear whether nurses who did data collection phone calls after 30 days were blinded for randomization.  Statistician was not blinded. | Loss to follow-up was limited (<1%), equally divided over groups and reasons for missing data were described.  Many eligible patients not included, due to decline or not being reached. | Research protocol published in advance. Methods are followed and expected outcomes reported as planned. | Single center  Many outcomes were self-reported by patients.  Participation bias not excluded as number of hospital admissions in both groups lower than expected.  After all underpowered study due to lower number of hospital admissions than predicted.  Patients and caregivers who did not pass the mental cognition screening examination were excluded. |

ED: Emergency department
